# Abnormal Baseline Brain Activity in Non-Depressed Parkinson’s Disease and Depressed Parkinson’s Disease: A Resting-State Functional Magnetic Resonance Imaging Study

**DOI:** 10.1371/journal.pone.0063691

**Published:** 2013-05-22

**Authors:** Xuyun Wen, Xia Wu, Jiangtao Liu, Ke Li, Li Yao

**Affiliations:** 1 State Key Laboratory of Cognitive Neuroscience and Learning, Beijing Normal University, Beijing, China; 2 School of Information Science and Technology, Beijing Normal University, Beijing, China; 3 Beijing Xuan Wu Hospital, Beijing, China; 4 Beijing 306 Hospital, Beijing, China; Institute of Psychology, Chinese Academy of Sciences, China

## Abstract

Depression is the most common psychiatric disorder observed in Parkinson’s disease (PD) patients, however the neural contribution to the high rate of depression in the PD group is still unclear. In this study, we used resting-state functional magnetic resonance imaging (fMRI) to investigate the underlying neural mechanisms of depression in PD patients. Twenty-one healthy individuals and thirty-three patients with idiopathic PD, seventeen of whom were diagnosed with major depressive disorder, were recruited. An analysis of amplitude of low-frequency fluctuations (ALFF) was performed on the whole brain of all subjects. Our results showed that depressed PD patients had significantly decreased ALFF in the dorsolateral prefrontal cortex (DLPFC), the ventromedial prefrontal cortex (vMPFC) and the rostral anterior cingulated cortex (rACC) compared with non-depressed PD patients. A significant positive correlation was found between Hamilton Depression Rating Scale (HDRS) and ALFF in the DLPFC. The findings of changed ALFF in these brain regions implied depression in PD patients may be associated with abnormal activities of prefrontal-limbic network.

## Introduction

For people with Parkinson’s disease (PD), depression is the most common and disabling symptom, and up to 50 percent of people with PD experience mild or moderate depressive symptoms [1], [2]. In addition to the unpleasant mood characteristics, depression can worsen the symptoms of PD, such as motor symptom deterioration [3], [4], rapid disease progression [5] and cognitive attenuation [3], [6]. Therefore, understanding and characterizing the underlying brain mechanisms of depression in PD patients using a neuroimaging approach is clearly an international imperative.

During the last decades, the pathophysiology of depression in PD patients has been accumulated from structural and functional neuroimaging studies [2], [7], [8], [9], [10]. High-resolution structural magnetic resonance imaging (MRI) showed PD patients with depression displayed abnormality in size of some areas, including the orbitofrontal gyrus, the superior temporal pole, and the mediodorsal thalamus, when compared with the patients with PD alone [2], [9]. Functional neuroimaging techniques were also been used to study depression in PD patients [2], [7]. A previous PET study found decreased levels of regional cerebral blood flow (rCBF) in the medial prefrontal cortex and the cingulated cortex in depressed PD group contrast to non-depressed PD group [7]. Recently, Cardoso and his colleagues using functional magnetic resonance imaging (fMRI) observed decreased activities in the left mediodorsal thalamic nucleus and the left dorsomedial prefrontal cortex of depressed PD patients but not of non-depressed PD patients [2]. These abnormal brain regions, which were found in these previous studies, mainly focused on the prefrontal cortex and limbic system, implying depression in PD patients may be associated with abnormal alterations in the prefrontal-limbic network.

Recently, resting-state fMRI has been widely used for investigating the brain functions under normal and pathological conditions for several special advantages, including high-resolution, no radiation use, and easy application [11], [12], [13], [14]. During rest, low-frequency blood-oxygen level fluctuations within a specific frequency range (0.01–0.08 Hz) are considered to be related to spontaneous neuronal activity [11], [12], [15]. The amplitude of low-frequency fluctuations (ALFF), in a method developed by Zang et al., has been widely applied to explore abnormal brain activity associated with some neuropsychiatric disorders, including mild cognitive impairment (MCI) [16], depression [17], Alzheimer’s disease (AD) [18], schizophrenia [19] and medial temporal lobe epilepsy [20]. Compared with traditional, task-related fMRI, the resting-state fMRI can be performed in all manner of people and is especially fit for people who are unable to cooperate with functional tasks [21]. To date, few resting-state fMRI studies have examined whether depressed PD patients present an abnormal activities.

In our study, we utilized ALFF to investigate the alterations in resting state brain activities in depressed PD patients compared with non-depressed PD patients. These abnormalities may be a trait marker and could be helpful for the future diagnosis of depression in PD patients. Based on previous studies, we hypothesized that an abnormal ALFF would be discovered in certain areas of the prefrontal-limbic network in depressed PD patients contrast to those patients with PD alone. In addition, we also compared PD patients those with and without depression with normal controls (Ncs).

## Methods

### Ethics Statement

The human fMRI experiment conducted in this study was approved by the Institutional Review Board of Beijing Normal University (BNU) Imaging Center for Brain Research, National Key Laboratory of Cognitive Neuroscience. All of the subjects gave written informed consent according to the guidelines set by the MRI Center of Beijing Normal University.

### Participants

Twenty-one right-handed NCs and thirty-three right-handed patients with idiopathic Parkinson’s disease, who were recruited from the Beijing Xuan Wu Hospital of China, participated in this study after giving written informed consent. The diagnosis of PD was based on medical history, physical and neurological examinations, response to levodopa or dopaminergic drugs, and laboratory tests and MRI scans to exclude other diseases. All subjects came in off medication for imaging and neuropsychological testing. Only PD patients with normal cognitive function as defined by a score on the Mini-Mental State Examination (MMSE) of 27 or more [22] were selected. Seventeen of PD patients were diagnosed with depression disorder according to the Diagnostic and Statistical Manual of Mental Disorders, 4th edition (DSM-IV) criteria (American Psychiatric Association, 1994) and the remaining sixteen patients had PD alone. The 24-item Hamilton Depression Rating Scale (HDRS) was used to evaluate the severity of depression and all depressed PD patients had a score of at least 8 points at HDRS [23]. Additionally, Unified Parkinson’s Disease Rating Scale (UPDRS) [24] and Hoehn and Yahr (HY) [25] were also recorded for describing the severity of the PD. The detailed clinical data were shown in Table I.

**Table 1:** Clinical and demographic characteristics.

Index	Depressed	non-depressed	normal
Age (years)	64.4±13.4	60.7±18.7	55.4±16.4
Time since diagnosis(years)	6.4±5.4	5.6±7.4	0
Hamilton scale	15.2±7.8	4.4±4.4	–
HY	2.1±1.9	1.5±1	–
UPDRS	42±46	33.8±24.2	–
MMSE	29.5±0.5	29.2±2.2	–
Gender (male/female)	7/10	8/8	13/8

Note: Abbreviations: HY–Hoehn and Yahr; UPDRS–Unified Parkinson’s Disease Rating Scale; MMSE–Mini Mental state examination.

### fMRI Data Acquisition

All fMRI data were acquired on a 3-Tesla Siemens whole-body MRI system scanner at Xuan Wu Hospital in Beijing, China. Foam padding and earplugs were used to limit head movement and reduce scanner noise for the subject. During the scan, the subjects were instructed to rest and keep their eyes closed without thinking about anything in particular. The functional images were collected using echo planar imaging (EPI) sequence. For each subject, 210 images were collected and the imaging parameters were as follows: repetition time = 2000 ms; echo time = 40 ms; Flip Angle = 90^o^; slice = 28; matrix size = 64×64; voxel size = 4×4×5 mm^3^. A high-resolution, three-dimensional T1-weighted structural image was acquired for each subject with the following parameters: repetition time = 2100 ms; echo time = 3.25 ms; Flip Angle = 10^o^; slice = 176; matrix size = 224×256; voxel size = 1×1×1 mm^3^.

### Data Processing

Image preprocessing was performed using Statistical Parametric Mapping (SPM8, http://www.fil.ion.ucl.ac.uk/spm). Allowing for the equilibration of the magnetic field, the first 10 volumes were discarded. The remaining 200 time points were slice-timing corrected to the middle axial slice, and all images were then realigned to the first image to account for head motion. A participant would be excluded if the translation and rotation parameters exceeded ±2 mm or 2^o^ during the whole fMRI scans. In our study, no subjects were excluded. After slice acquisition and head motion correction were performed, all of the volumes were spatially normalized to the standard SPM8 Montreal Neurological Institute (MNI) template, re-sampled to 3 mm cubic voxels, and smoothed by a Gaussian kernel with the full width set at a half maximum of 5 mm.

**Table 2:** Brain regions exhibiting an altered ALFF between depressed PD patients and non-depressed PD patients.

Brain regions	L/R		Cluster size		BA	MNI coordinate			T value
						*x*	*y*	*z*	
*Depressed PD patients>non-depressed PD patients*									
Medial prefrontal cortex		L&R		59	10	0	51	−3	4.19
Anterior cingulated cortex						−3	51	1	3.55
Middle frontal gyrus		R		27	9	54	24	33	4.42
Temporal lobe		L		22	–	−45	−39	−6	3.89
Superior frontal gyrus		R		17	8	24	33	54	3.85
Middle frontal gyrus		R		16	10/46	42	39	24	3.95
*Depressed PD patients<non-depressed PD patients*									
Cerebellum posterior lobe		R		38	–	36	−63	−36	3.27
Cerebellum posterior lobe					–	51	−69	−45	3.56
Cerebellum posterior lobe						42	−69	−36	3.1
Cerebellum anterior lobe		L&R		17	–	−3	−42	−6	3.48
Cerebellum anterior lobe						0	−51	−3	2.87

BA: Brodmann area; MNI: Montreal neurological institute.

### ALFF Calculation

After preprocessing in SPM8, Further data preprocessing and ALFF analysis was performed with REST software (http://resting-fmri.sourceforge.net) [26]. Firstly, the linear trend was removed, and every voxel was band-pass filtered (0.01 Hz<f<0.08 Hz) to remove the effects of low-frequency drift and high-frequency noise. Then we removed the influence of head motion using linear regression but white matter and cerebral cerebrospinal fluid (CSF) were not regressed out. The ALFF calculation procedure: 1) Fast Fourier Transform (FFT) was used to convert all voxels from the time domain to the frequency domain; 2) the ALFF of every voxel was calculated by averaging the square root of the power spectrum across 0.01 Hz to 0.08 Hz; 3) the resulting ALFF was converted into z-scores by subtracting the mean and dividing by the global standard deviation for standardization purposes.

**Table 3:** Brain regions exhibiting an altered ALFF between non-depressed PD patients and NCs.

Brain regions	L/R	Cluster size	BA	MNI coordinate			T value
				*x*	*y*	*z*	
*Non-depressed PD patients<NCs*							
Caudate	L	483	–	−18	9	21	6.31
Putamen				−21	6	15	3.06
Caudate	R	477	–	19	5	24	4.8
Thalamus				9	−4	7	3.92
SMA	R	52	6	3	12	69	4.56
Superior frontal gyrus			–	6	−3	78	3.23
Vermis 3	L&R	38	–	3	−42	−3	4.56
Posterior cingulate gyrus	L	20	31	−18	−48	33	3.44
*Non-depressed PD patients>NCs*							
Medial prefrontal gyrus	L	36	10	−3	63	−3	3.36
Angular	R	81		51	−51	36	4.42
Supramarginal				45	−39	42	3.68
Middle temporal gyrus	L	50		−54	−30	−18	3.91
Middle occipital gyrus	R	31	19	39	−84	21	3.81
Inferior frontal gyrus	L	24		−45	27	3	3.81
Precuneus	L	30	31	−6	−60	24	3.92
Calcarine				−9	−69	18	3.13
Superior occipital gyrus	L	16	7	−18	78	42	3.85
Superior occipital gyrus			19	−21	−87	36	3.2
Superior occipital gyrus	R	30	19	21	−87	33	3.82

BA: Brodmann area; MNI: Montreal neurological institute; SMA: supplementary motor area.

### Statistical Analysis

A two-sample t-test was performed to explore the ALFF differences among depressed PD patients, non-depressed PD patients and NCs. The between-group statistical threshold was set at p = 0.005 and cluster size> = 432 mm^3^ (16 voxels), which corresponded to a corrected p<0.05. This correction was determined by the Monte Carlo simulations, which were performed with REST software (http://resting-fmri.sourceforge.net) (whole brain mask: 70831 voxels; simulation number = 5000) [26].

**Table 4:** Brain regions exhibiting an altered ALFF between depressed PD patients and NCs.

Brain regions	L/R	Cluster size	BA	MNI coordinate			T value
				*x*	*y*	*z*	
*Depressed PD patients<NCs*							
Caudate	L	661	–	−18	9	21	6.42
Putamen			–	−20	4	15	2.8
Caudate	R	469	–	15	−6	−18	4.5
Precuneus	R	21	–	3	−48	72	3.98
Precuneus	L		–	−3	−54	69	3.11
Superior frontal gyrus	R	29	9	36	45	36	3.21
Middle frontal gyrus	R	16	6	42	0	57	3.61
Medial frontal gyrus	R	20	–	0	27	42	3.16
Superior temporal gyrus	R	37	38	48	18	−12	3.28
Thalamus	R	19	–	3	−18	9	3.61
*Depressed PD patients>NCs*							
Angular gyrus	R	45	–	48	−63	33	3.49
Middle temporal gyrus	L	180	21	−57	−21	−6	4.64
Middle temporal gyrus	R	81	21	66	−39	−3	5.25
Inferior frontal gyrus	L	35	–	−45	27	−6	4.67
Precuneus	L	29	–	−33	−75	39	4.11
Inferior parietal gyrus	L	29	40	−66	−33	33	4.05
Fusiform gyrus	R	45	–	60	−18	−27	4.24

BA: Brodmann area; MNI: Montreal neurological institute.

### Correlation between Clinical Data and ALFF

To examine the association of the ALFF abnormality with the severity of the depression of PD patients, we performed a partial correlation analysis (controlling age and gender) between HDRS data and ALFF values extracted from clusters of voxels, which showed the most significant differences between depressed and non-depressed PD patients. Each of the clusters was the intersection of the corresponding region defined by Anatomical Automatic Labeling atlas toolbox [27] and the within group two sample t-test map with a cut-off threshold at p = 0.005.

## Results

### Clinical and Demographic Testing of Samples

In regard to our clinical and demographics of sample participants (Table I), there were no significant differences in gender (t = 0.495, p = 0.624), age (t = 1.668, p = 0.105), MMSE (t = 0.692, p = 0.495), HY (t = 0.394, p = 0.730) and UPDRS scores (t = 1.656, p = 0.110) between depressed PD patients and non-depressed PD patients. As for HDRS (t = 8.965, p<0.001), PD patients with depression were significantly higher than those with PD alone. Our study tested non-depressed PD patients and NCs and found the differences in gender (t = 0.709, p = 0.483) and age (t = 1.414, p = 0.166) were also not significant.

### Depressed PD Patients versus Non-depressed PD Patients

**Figure 1:**
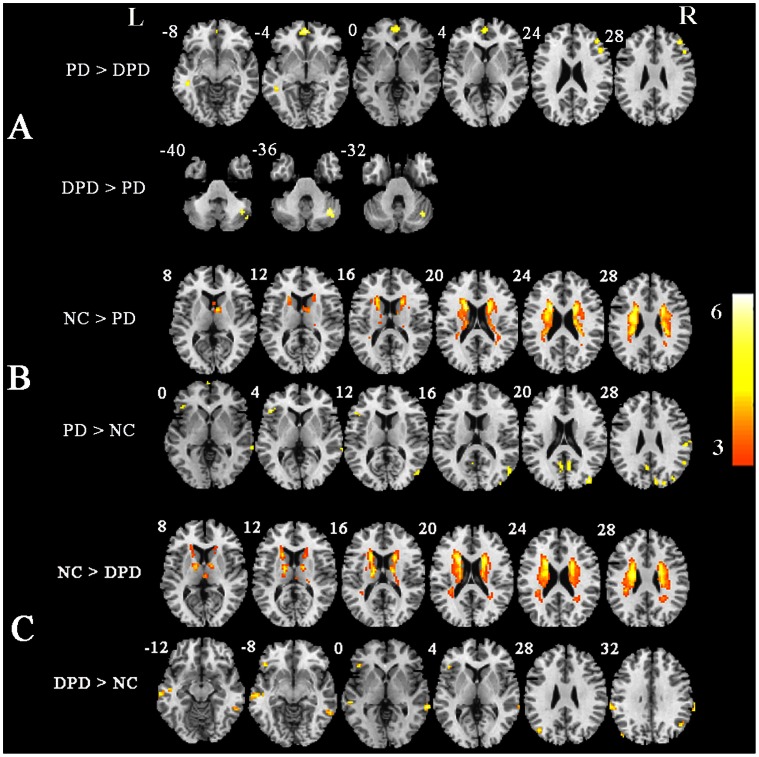
Statistical parametric map showing the significant differences in the ALFF between three groups: depressed PD patients, non-depressed PD patients and NCs. A) The differences between depressed PD patients and non-depressed PD patients. B) The differences between non-depressed PD patients and NCs. C) The differences between depressed PD patients and NCs. The threshold for display was set to p<0.005, cluster size> = 432 mm^3^.

Compared to non-depressed PD patients, depressed PD patients exhibited a decreased ALFF in the right dorsolateral prefrontal cortex (DLPFC), ventromedial prefrontal cortex (vMPFC), the rostral anterior cingulate cortex (rACC), the superior frontal cortex and the right middle temporal gyrus. The opposite (depressed>non-depressed) was observed in the right cerebellum posterior lobe and the right cerebellum anterior lobe. Detailed information about the Montreal neurological institute (MNI) coordinates and clusters was provided in Fig. 1A and Table II.

### Non-depressed PD Patients versus NCs

The group differences between non-depressed PD patients and NCs were shown in Fig. 1B and Table III. The ALFF in NCs was higher than in patients with PD alone in the bilateral caudate, the left putamen, the supplementary motor area (SMA), the bilateral superior frontal gyrus and the posterior cingulated gyrus. The ALFF, which was significantly lower in NCs than non-depressed PD, was found in the left middle temporal gyrus, the right middle occipital gyrus, the bilateral superior occipital gyrus, the left inferior temporal gyrus, the left precuneus and the right angular gyrus.

### Depressed PD Patients versus NCs

As shown in Fig. 1C and Table IV, the depressed PD group demonstrated a decreased ALFF in the bilateral caudate, the left putamen, the bilateral precuneus, the right superior frontal gyrus, the right middle frontal gyrus, the left putamen, the right medial frontal gyrus, the right superior temporal gyrus and the right thalamus contrast to NCs. Conversely, the right angular gyrus, the bilateral middle temporal gyrus, the left inferior frontal gyrus, the left precuneus, the left inferior parietal gyrus and the right fusiform gyrus displayed an increased ALFF in the depressed PD patients.

### Correlations between ALFF Values and HDRS

We examined the relationships between the HDRS and ALFF in regions with significant group differences (depressed PD patients vs. non-depressed PD patients), including DLPFC, rACC, vMPFC. The only significant correlation we found between ALFF values and HDRS was in the DLPFC (r = 0.698, p = 0.003). The other correlation were all less than ±0.2 (p>0.05).

## Discussion

The present fMRI study aimed to investigate the alterations in resting-state brain activities in depressed PD patients, and we found a decreased ALFF in the DLPFC, the vMPFC and the rACC in depressed PD patients when compared with non-depressed PD patients. Inversely, An increased ALFF (depressed PD patients> non-depressed PD patients) was observed in the cerebellum posterior cortex. In addition, when compared with NCs, the depressed PD patents and non-depressed PD patients both showed altered activities mainly in the basal ganglia and the prefrontal cortex. Furthermore, a significant positive correlation was found between the HDRS score and ALFF within the DLPFC.

The DLPFC provides a key hub in the prefrontal-limbic network which connects to the orbitofrontal cortex, the thalamus, parts of the basal ganglia, the hippocampus, and primary and secondary association areas of the neocortex [28]. It has an important role in cognitive, executive and emotional processes, especially the down-regulation of negative emotional conditions [29], [30], [31]. Abnormal activity in the DLPFC may lead to a cognitive and mental disorder and partly contribute to interest or pleasure deficiency and cognition declines exhibited by patients with depression [32], [13]. Our current study using resting-state fMRI found a decreased ALFF in the DLPFC in depressed PD patients contrast to those patients with PD alone and a positive correlation was also been found between HDRS score and ALFF values in the DLPFC. Consistent with our result, the hypoactivity in the DLPFC in depression has been identified by many previous studies, which was regarded as a critical hallmark for depression [7], [32], [33], [34], [35], [36], [37]. For example, Bench et al. found a decreased rate of metabolism and decreased rCBF levels in the DLPFC in depression [32], and an increase in activity in the DLPFC will remit depression symptoms [37]. Similar results were also been found in depressed PD groups. A previous PET study reported a decreased rCBF level in the DLPFC of depressed PD patients compared with non-depressed PD patients [7], and stimulating the DLPFC with repetitive transcranial magnetic stimulation (rTMS) can be effective in remitting depression symptoms in PD [33], [35]. Together with these findings, we speculated that hypoactivation in the DLPFC may be an important factor for the genesis and development of depression in PD patients.

The vMPFC seems to be a critical area in PD-associated depression. The abnormalities within the vMPFC in patients with major depressive disorder (MDD) have been documented in previous structure and functional studies [38], [39], [40]. In our study, we using ALFF investigated the abnormalities of depressed PD patients and found a decreased activity in the vMPFC in depressed PD contrast to those without depression PD patients. Similar to our finding, a previous PET study compared the regional blood flow of depressed and non-depressed PD patients and found a decreased rCBF level in the vMPFC [7]. The vMPFC is connected with the ACC, the hippocampus and the amygdala [41] and plays a vital role in emotion generation and regulation [42], [43]. The activity of the vMPFC was associated with the suppression of affective responses to a negative emotional signal and might dampen amygdala activity [44]. Jonestone et al. found, during an effortful affective reappraisal task, normal subjects showed an inverse relationship between amygdala but depressed individuals were not [45]. Therefore, the decreased level of activity in the vMPFC in depressed PD patients may lead to an imbalance in the inhibitory influence of the amygdala on activity leading to the genesis of depression. Our data do not allow us to state that an altered relationship between the vMPFC and the amygdala is responsible for the observed decrease in activity levels in the vMPFC, but, based on previous reports, this hypothesis should be evaluated in the future.

In addition, a decreased ALFF in the rACC was also reported in our current study. The rACC is a part of the brain’s limbic system which is strongly connected with the amygdala, the orbitofrontal cortex and the hippocampus, and it has been reported to be associated with the processing and integration of affect-related information [46], [47]. Lesions in the rACC can lead to a series of symptoms, including apathy, inattention, the dysregulation of autonomic functions, akineticmutism and emotional instability, which overlap considerably with the quintessential symptoms of patients with MDD, implying depression has a relationship with abnormal activity in rACC [48]. A recent resting-state fMRI study also demonstrated that the severity of depression in PD patients was correlated with the ALFF values in the rACC, which was consistent with the result of our current study [49]. However, in the absence of normal control group, Skidmore and his colleagues’ study could not decide whether the activity in rACC was decreased or increased in depressed PD patients. Our study compensated for this limitation and identified the rACC showed a decreased ALFF in depressed PD patients compared with non-depressed PD group, which gave a more complete fMRI status of depression in PD patients.

The regions we found to have a decreased ALFF in the depressed PD group, including the DLPFC, the vMPFC and the rACC, are parts of the prefrontal-limbic network, which is important for affective processing [50]. Previous non-invasive brain imaging study has identified abnormal changes in the prefrontal-limbic network existed in patients with MDD [41]. In our study, we found abnormal activity levels in prefrontal-limbic network were also existed in the depressed PD patients giving a new clue to the pathophysiology of depression in PD group.

In contrast to the decreased activities in the prefrontal-limbic network, we observed an increased ALFF in the right cerebellum posterior lobe in depressed PD patients compared with non-depressed PD patients. The traditional view of the cerebellum is that it is only responsible for the regulation of motor functions, but recent studies identified this area also being associated with emotional and cognitive processing [51], [52]. Previous studies demonstrated the patients with depression showed abnormal changes in cerebellum [53], [54], [55], [56]. Pillay et al. reported that patients with depression showed a volume reduction in the cerebellum [54]. Using fMRI, Liu et al. and Guo et al. found a decrease in regional homogeneity (ReHo) in depression patient group compared with NCs [55], [56]. Additionally, the reciprocal connections linking the cerebellum with brainstem areas contain neurotransmitters involved in mood regulation, including serotonin, norepinephrine and dopamine [57]. The degeneration of the dopaminergic pathway, a hallmark of PD patients [8], may lead to the genesis of increased activity in the cerebellum. Our current study provides evidence for the involvement of cerebellar abnormality in depressed PD patients.

Additionally, comparing ALFF maps between non-depressed PD patients and NCs, our study also investigated the PD related pathophysiology and found the altered activities in PD mainly focused on basal ganglia (including putamen, caudate) and prefrontal cortex. Findings from previous studies suggested that basal ganglia plays an important role in cortico-subcortical circuits, including motor, oculomotor, dorsolateral, prefrontal, lateral orbitofrontal and anterior cingulate [58], [59]. PD is a movement disorder characterized by the triad of bradykinesia, tremor at rest and muscular rigidity [60], [61], which mainly result from varying forms of abnormally patterned activity throughout the motor circuit [62]. Similar to our study, some recent researches using the index of ALFF also found abnormal activities in PD patients mainly in prefrontal cortex and motor cortex including SMA, the mesial prefrontal cortex and middle frontal cortex [63], [64]. Combined these previous findings with our current study, the speculation, that PD was associated with abnormal changes in motor circuit, was further been demonstrated.

It has recently been reported that in-scanner head motion can have an influence on analysis results even though traditional realignment was performed [65], [66]. In our study, to control the impact of head motion, we not only made every effort to reduce its occurrence in the scanner and precluded those subjects with the translation and rotation parameters exceeded ±2 mm or 2^o^ during the whole fMRI scan, but also removed the influence of head motion using linear regression based on REST software (http://resting-fmri.sourceforge.net) [26] before ALFF calculating. In addition, following previous studies, the mean relative displacement was used to measure subjects’ head motion in scanner [65], . Then two sample t-test was used to test differences of head motion between groups and no significant differences were found (depressed PD patients vs non-depressed PD patients: t = 0.435, p = 0.666; non-depressed PD patients vs NCs: t = 0.361, p = 0.720; depressed PD patients vs NCs: t = 0.795, p = 0.432). The significant correlations between ALFF and mean relative displacement were also not found (ACC: r = −0.114, p = 0.662; MPFC: r = −0.017, p = 0.947; DLPFC: r = 0.223, p = 0.39) according to calculating the correlations coefficients between these two indexes. These findings suggested the significant differences among groups in our current study may have no relationship with head motion. However, further works are needed to explore this issue.

In summary, our study used ALFF to examine the alterations in the resting state between depressed PD patients and non-depressed PD patients and found abnormal neural activity levels in several brain areas associated with the prefrontal-limbic network. Our study not only advances the knowledge of depression in PD but also provides a new insight into the underlying neural mechanism behind the high rate of depression in PD patients.
